# Network stiffness: A new topological property in complex networks

**DOI:** 10.1371/journal.pone.0218477

**Published:** 2019-06-18

**Authors:** Dimitrios Tsiotas

**Affiliations:** Department of Planning and Regional Development, University of Thessaly, Pedion Areos, Volos, Greece; Asia Pacific Center for Theoretical Physics, REPUBLIC OF KOREA

## Abstract

Aiming at serving the interdisciplinary demand in network science, this paper introduces a new concept for complex networks, named network stiffness, which is extracted from structural engineering by assuming that a complex network behaves similarly with a structured framework. This analogy allows interpreting that a complex network can resist against any cause attempting to induce deformation changes to the network’s structure, regardless of whether the network is material or not. Within this framework, this paper examines the context of applying the conceptual analogy of stiffness from the field of structural engineering to network science and then it develops computational approaches capturing different aspects of network stiffness so that to be used in complex network analysis. The implementation of these approaches to a real-world network (global inbound tourism network) shows that stiffness can produce interesting insights to complex network analysis about the factors related to changes caused to the structure and the status of a complex network.

## 1. Introduction

Research in complex networks had diachronically the merit to be multidisciplinary, which obviously contributed to the evolution of this scientific field into an emerging academic discipline, the so-called network science (NS) [1,2]. Provided that synthesis is a major perspective for multidisciplinary modeling, the synthetic approach has also been proven fruitful in the study of complex networks [3–5]. Some indicative examples are the conceptualization of the preferential attachment mechanism [5,6] by statisticians [7], which emerged from the study of species per genus of flowering plants, the conceptualization of the small-world phenomenon by sociologists [8], which was introduced during social experiments of forwarding mails worldwide along groups of personal connections, the conceptualization of spatial networks from physicists and geographers [4], the conceptualization of visibility graphs by applied mathematicians [9], who introduced a method of transforming a time-series into a complex network, and many others.

Aiming at serving the multidisciplinary demand in NS, this paper introduces a new concept named “*network stiffness*”, which is extracted from structural engineering by assuming that a complex network behaves similarly with a structured framework, in order to be used in the study of complex networks. In terms of structural engineering [10], stiffness is the property expressing the level of consolidation of a structural framework and, thus, expressing the framework’s resistance against any cause inducing deformation and structural changes. Stiffness is a well-established concept in the field of structural engineering [11], which is used within the context of structural analysis and design in order to measure the level of deformation that sets-of-loads induce to structured elements. Mostly, stiffness is used for the computation of the geometric and structural properties of buildings and other construction works, in order these structures to be static-adequate and resistant [10,11].

Considered as a term related to networks, stiffness has been used to describe polymer and biological networks. Among the few relevant papers existing in the literature, a prime reference can be found back in the authors of [12], who studied stiffness of rats’ cardiovascular collagen networks, aiming at measuring the accumulation of collagen and its structural remodeling and afterwards at correlating their findings with the behavior of the rats’ myocardium. In the work of [13], the authors used the term for rigid rods polymer networks in order to study the elasticity of a two-dimensional random network of such polymer in a model incorporating the rods’ anisotropic elasticity and the random geometry of the network. In a later paper, the author of [14] studied stiffness of spring networks aiming at relating the viscoelastic response of these networks with some critical structural measures, such as the proximity to isostaticity, the shear-modulus, and the creep. In the most recent work of [15], the authors studied stiffness of collagen-I and alginate interpenetrating networks aiming at investigating whether tuning the stiffness of a model wound dressing biomaterial could control the behavior of dermal fibroblasts.

As being evident by these few available papers, in the current literature, stiffness is a concept related to networks that are explicitly considered as material bodies (structures) and not as graph models. Therefore, the concept is restricted to the material nature of networks and not to their state of connectivity. Within this context, this paper attempts to provide a broader conceptualization of network stiffness, aspiring to be applicable to the total family of complex networks, regardless they are material or not. In particular, it attempts to link the concept of network stiffness with the state of connectivity and thus to link this concept with the topology of complex networks. Such linkage is possible by assuming that a complex network behaves similarly with a structured framework, even in cases that network links (edges) are of immaterial nature. This assumption allows computing in complex networks either the forces applied to network nodes, when the effect of forces is measurable, or the deformations (displacements) caused to network nodes by known forces, in accordance with the existing methodology of design and analysis of structural frameworks (e.g. beams, columns, structural plates) in structural mechanics [10,11]. Such computations can provide insights about the response-mechanism of complex networks against structural changes and thus they can be useful in the relevant research of network dynamics.

Currently, the common approach of measuring the perturbations and their propagation in complex networks was proposed by the authors of [16]. In particular, for a given activity (node-attribute) *X*, which for the nodes *i* and *j* takes respective values *X* = *x*_*i*_ and *X* = *x*_*j*_, the authors used a correlation matrix defined by the permanent perturbations *dx*_*j*_ on the value *x*_*i*_ and *dx*_*j*_ on the value *x*_*j*_, according to the formula:

$$G_{ij}(X)=\left|\frac{dx_{i}/x_{i}}{dx_{j}/x_{j}}\right|,$$ (1)

This correlation matrix enjoys lots of applications, mainly in biology, and it generally captures the influence of node *j* on *i*. [16,17]. Based on relation (1), the authors of [16] defined a measure named impact of node *i*, according to the formula:

$$I_{i}(X)=\sum_{j=1}^{n}A_{ij}G_{ij}^{T},$$ (2)

where *A*_*ij*_ is the adjacency matrix of the network and $G_{ij}^{T}$ is the transposed matrix of *G*_*ij*_. This measure captures the average response of the node’s (*i*) neighborhood to the perturbation of *i* [16].

At next, the authors defined a measure named *stability* of node *i*, according to the formula:

$$S_{i}(X)={\left(\sum_{j=1}^{n}A_{ij}G_{ij}\right)}^{-1},$$ (3)

which, loosely, has an inverse configuration in comparison with *I*_*i*_. However, a critical difference with *I*_*i*_ is that node stability is defined by the original *G*_*ij*_ matrix instead of by the transposed $G_{ij}^{T}$ and thus the interpretation of *S*_*i*_ is different than the inverse of *I*_*i*_. In particular, node stability captures the inverse response of node *i* to individual perturbations of its nearest neighbors [16]. Based on the method of analogy, the new concept of network stiffness seems capable to provide insights in the study of complex networks dynamics. Toward this direction, the computational approaches developed in this paper are evaluated in comparison with the measures proposed by the author of [16].

The remainder of this paper is organized as follows: section 2 presents the methodological framework of the study; it configures the conceptual analogy of stiffness between a structured framework and a complex network and it develops computational approaches of network stiffness in complex network analysis. Section 3 presents a couple of implementations of the proposed concept to a real-world network of global inbound tourism flows and it evaluates the proposed computational approaches in comparison with established propagation measures in complex networks. Section 4 discusses limitations of the study and addresses for further research, and finally, in section 5, conclusions are given.

## 2. Methodological framework

### 2.1. The conceptual analogy of stiffness: From structural engineering to complex networks

**Fig 1:**
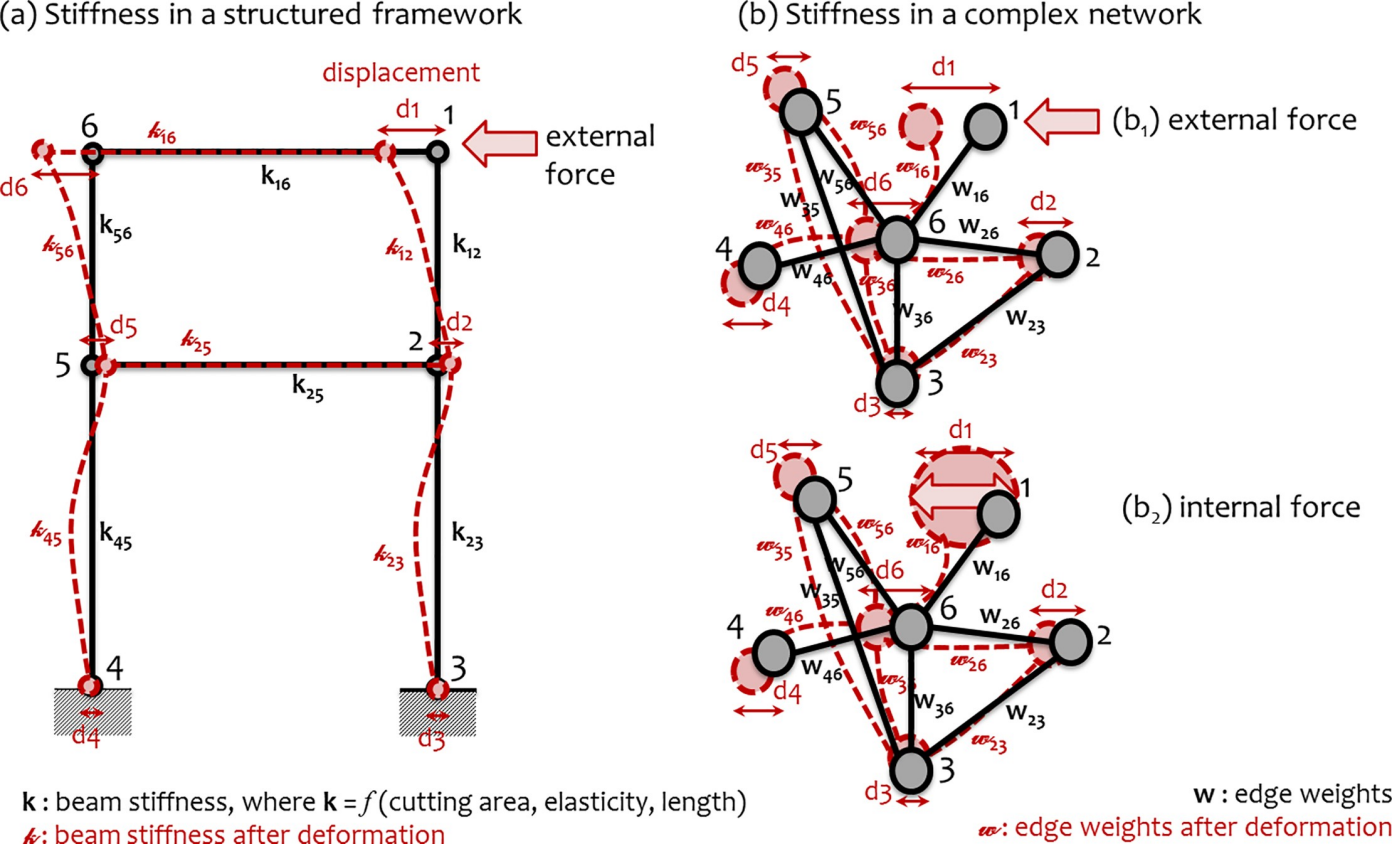
Semantic diagram for the conceptual analogy of stiffness, from the structural engineering to complex networks: (a) in structural engineering, stiffness describes the ability of a structured framework to resist against an external force, whereas (b) in complex networks, stiffness represents the network’s ability to resist against (b_1_) external or (b_2_) internal causes (forces) applied to network nodes inducing changes in the network.

In structural engineering, stiffness is the property expressing the resistance of a structured framework against any force attempting to cause structural changes [10]. The measure of stiffness is also related with the ease with which an external force is propagated along the body of the structured framework and it depends on the geometry (cutting area, length) and on the composition (elasticity) of the elements (beams, columns) composing the framework [10,11]. Based on the method of analogy, we can assume that a complex network behaves similarly with a structured framework and thus we can transfer the concept of stiffness from the field of structural engineering to complex networks (Fig 1). This is possible because, first, both a structured framework and a complex network can enjoy discrete modeling into a graph composed by sets of nodes and edges (links). In particular, in a structured framework [10,11], the structural elements (i.e. beams or columns) can be modeled into edges and the edge intersections can be modeled into nodes. Similarly, a complex network is by default defined by a graph model composed by a set of nodes and links [4,6].

Second, in a complex network, edge weights (*w*_*ij*_) express the power of connectivity between nodes [3,4] and thus they configure the network’s capability to spread information along the network structure, regardless of whether the network is material or not. Similarly, the elasticity moduli (*k*_*ij*_) of the structural elements (*ij*) define the capability of a structured framework to distribute the effect of external forces throughout the framework’s body [10] and thus they configure the power of connectivity between nodes. Third, the stiffness ***k*** of a structured framework is modeled into a square matrix *K*_*n*×*n*_ (called stiffness matrix or stiffness tensor), with length equal to the number of nodes (intersections) (*n*) in the framework [10,11]. The stiffness matrix includes the elastic moduli *K*_*n*×*n*_ = [*k*_*ij*_] of the structural elements configured by nodes *i* and *j* [10] and thus it expresses a connectivity matrix *W*_*n*×*n*_ = [*w*_*ij*_] of the framework’s graph model, in which edge weights are *w*_*ij*_ = *k*_*ij*_.

These similarities configure the analogy between a structured framework and a complex network and, therefore, they allow assuming that the notion of stiffness of a structured framework (e.g. of a beam structure) can be used as an aspect of network connectivity or topology. Based on this analogy, the next section develops a pair of computational approaches of network stiffness in complex network analysis.

### 2.2. Network stiffness: Developing computational approaches

#### 2.2.1. Vectors of forces and displacements

Considering network stiffness as a topological property of complex networks allows computing (in a complex network) either the forces applied to network nodes, when the effect of forces is measurable, or the deformations (displacements) caused to network nodes by known forces, in accordance with the existing methodology in structural mechanics. Based on the analogy which was previously described, we can assume the weights matrix *W*^*n*×*n*^ = [*w*_*ij*_] of a complex network as the stiffness matrix *K*^*n*×*n*^ = [*k*_*ij*_] of a structured framework. Let’s also consider a node-attribute (or activity) *X*, measured at two different times *X = x*(*t*_*a*_) and *X = x*(*t*_*b*_) and let’s assume that the change *x*(*t*_*b*_)–*x*(*t*_*a*_) observed for this attribute can be seen as the displacements (**d** = d(*t*_*b*_)–d(*t*_*a*_) = *x*(*t*_*b*_)–*x*(*t*_*a*_)) caused by an external (or internal) force **f** (see Fig 1). Then we can compute the force applied to the set of network nodes *V*_*G*_ according to the relation [10,11]:

$$f^{n\times 1}=K^{n\times n}\cdot d_{\mathrm{observed}}^{n\times 1}\overset{\left(K^{n\times n}={\left[k_{ij}\right]}^{n\times n}\equiv W^{n\times n}={\left[w_{ij}\right]}^{n\times n}\right)}{=}W\cdot d_{\mathrm{observed}}=W\cdot {\left[d_{i}\left(t_{b}\right)–d_{i}\left(t_{a}\right)\right]}^{n\times 1},$$ (4)

where *n* is the number of network nodes. In structured frameworks, the forces vector **f**^*n*×1^ = [*F*_*i*_] is measured in force units, whereas, in complex networks, forces are measured in *w·x* (weights*·*node-attribute) units.

In contrast, by considering that a known force **f** is applied to the set of network nodes *V*_*G*_, we can compute the effect of this force (i.e. the displacements vector **d**^*n*×1^ = (d_1_, d_2_,…, d_*n*_)΄) on *V*_*G*_ by solving the linear system (4) and get:

$$d^{n\times 1}={\left(W^{n\times n}\right)}^{-1}\cdot f_{\mathrm{observed}}^{n\times 1},$$ (5)

where (*W*^*n*×*n*^)^−1^ is the inverse of the weights matrix *W*^*n*×*n*^ = [*w*_*ij*_]. A solution of relation (5) is possible when *W* is invertible matrix. In structured frameworks, the displacements vector **d** expresses the length of displacements along the direction of the external force **f** and it is measured in metric units. In complex networks, **d** expresses the effect of **f** on the network nodes and it is measured in *F*/*w* (force*/*weights) units.

According to relation (4), when **d** is a vector of ones (**d** = [1 1 1]΄) and the adjacency matrix *A*^*n*×*n*^ = [*a*_*ij*_] is the stiffness matrix (*K* = *A*), then the forces vector **f** equals to the network degrees **f**≡**k** = (*k*_1_, *k*_2_,…, *k*_*n*_). This observation implies that when the vector of network degrees is applied as force to an unweighted network (i.e. every node is subjected to a force equal to its degree) it causes unit effects (displacements). Further, it can be observed that when a single node is subjected to a force equal to its degree, the displacements caused to the other network nodes equal to the degree of the node where the force is applied. Similarly, when the vector of network strengths is applied as force **f** = **s** = (*s*_1_, *s*_2_,…, *s*_*n*_) to a weighted network it causes unit effects (displacements) to network nodes.

#### 2.2.2. The stiffness scale-factor

In structural engineering, network stiffness is the concept linking the vectors of forces (**f**) and displacements (**d**) in a cause-effect context [10,11]. This is because the stiffness matrix *K* is the tensor expressing a pair-wise relation of the form *K* = *f*(**f**,**d**). Within this framework, by assuming that a complex network behaves similarly with a structured framework, we can develop, in a complex network, a pair-wise property between any pair of node-attributes **x** and **y**, for which a cause-effect pattern of the form **y** = *f*(**x**) will exist (**x** = cause, **y** = effect). Within a structured-framework-alike context, this is possible by considering that one network attribute acts like the vector of forces (e.g. **y** = **f**) while the second acts like the vector of displacements (**x** = **d**).

In particular, let’s consider a complex network *G*(*V*,*E*), with stiffness *K*, and a pair of node-variables **x**,**y**, of length |*V*| = *n*. According to relation (4), node-variables **x**,**y** can attain a cause-effect relation within the context of network stiffness as follows:

$$f=K\cdot d\overset{\left(f\equiv x,d\equiv x\right)}{\Leftrightarrow }x=K\cdot y\overset{\left(\exists K^{-1}\right)}{\Rightarrow }y=K^{-1}\cdot x.$$ (6)

If we assume that the stiffness matrix is the connectivity matrix (*K*≡*W*), then relation (6) cannot be satisfied for the node-variables **x**,**y**. Therefore, we need to define the stiffness matrix in terms of *W*, namely *K = f*(*W*). To do so, let’s consider a vector **s** = [*s*_1_, *s*_2_, …,*s*_n_]΄ and the diagonal function diag(·) [18], as follows:

$$\mathrm{diag}\left(s\right)=\mathrm{diag}\left(\left[\begin{array}{c} s_{1}\\ s_{2}\\ \vdots \\ s_{n} \end{array}\right]\right)\equiv S_{d}(s)=\left[s_{ij}\right]=\left[\begin{array}{cccc} s_{1} & 0 & \cdots & 0\\ 0 & s_{2} & \cdots & 0\\ \vdots & \vdots & \ddots & \vdots \\ 0 & 0 & \cdots & s_{n} \end{array}\right]=\{\begin{array}{c} s_{i},\;i=j\\ 0,\;\mathrm{otherwise} \end{array}.$$ (7)

Then, we seek for a matrix *S*_d_(**s**) that produces the stiffness matrix *K*, according to the relation:

$$K=S_{d}(s)\cdot W.$$ (8)

Based on relation (8), the relation (3) gives:

$$x=K\cdot y\Leftrightarrow f=K\cdot d=(S_{d}\cdot W)\cdot d=\left[\begin{array}{cccc} s_{1}\cdot w_{11} & s_{1}\cdot w_{12} & \cdots & s_{1}\cdot w_{1n}\\ s_{2}\cdot w_{21} & s_{2}\cdot w_{22} & \cdots & s_{2}\cdot w_{2n}\\ \vdots & \vdots & \ddots & \vdots \\ s_{n}\cdot w_{n1} & s_{n}\cdot w_{n2} & \cdots & s_{n}\cdot w_{nn} \end{array}\right]\cdot d,$$ (9)

where **x** = [*x*_*ij*_]≡**f** = [*F*_*ij*_], **y** = [*y*_*ij*_]≡**d** = [d_*ij*_], and *W*_*n*×*n*_ = [*w*_*ij*_] is the connectivity (weights) matrix of network *G*. Equivalently to relation (9) we have:

$$\begin{array}{l} S_{d}{}^{-1}\cdot f=W\cdot d\Leftrightarrow \left[\begin{array}{cccc} 1/s_{1} & 0 & \cdots & 0\\ 0 & 1/s_{2} & \cdots & 0\\ \vdots & \vdots & \ddots & \vdots \\ 0 & 0 & \cdots & 1/s_{n} \end{array}\right]\cdot \left[\begin{array}{c} F_{1}\\ F_{2}\\ \vdots \\ F_{n} \end{array}\right]=\left[\begin{array}{cccc} w_{11} & w_{12} & \cdots & w_{1n}\\ w_{21} & w_{22} & \cdots & w_{2n}\\ \vdots & \vdots & \ddots & \vdots \\ w_{n1} & w_{n2} & \cdots & w_{nn} \end{array}\right]\cdot \left[\begin{array}{c} d_{1}\\ d_{2}\\ \vdots \\ d_{n} \end{array}\right]\Leftrightarrow \\ \Leftrightarrow \left[\begin{array}{c} F_{1}/s_{1}\\ F_{2}/s_{2}\\ \vdots \\ F_{n}/s_{n} \end{array}\right]=\left[\begin{array}{cccc} w_{11} & w_{12} & \cdots & w_{1n}\\ w_{21} & w_{22} & \cdots & w_{2n}\\ \vdots & \vdots & \ddots & \vdots \\ w_{n1} & w_{n2} & \cdots & w_{nn} \end{array}\right]\cdot \left[\begin{array}{c} d_{1}\\ d_{2}\\ \vdots \\ d_{n} \end{array}\right]={\left[\begin{array}{cccc} \sum_{i=1}^{n}w_{1i}d_{i} & \sum_{i=1}^{n}w_{2i}d_{i} & \cdots & \sum_{i=1}^{n}w_{ni}d_{i} \end{array}\right]}^{\prime }\Leftrightarrow \\ \Leftrightarrow s={\left[s_{i}=F_{i}/\sum_{j=1}^{n}w_{ij}d_{j},\;i=1,\ldots ,n\right]}^{\prime }or\;s={\left[x_{i}/\sum_{j=1}^{n}w_{ij}y_{j},\;i=1,\ldots ,n\right]}^{\prime } \end{array}.$$ (10)

According to this analysis, in the complex network *G*, **s** is the vector applied to the weights matrix *W* so that node-variable **y** (≡displacements vector **d**) to be the effect of node-variable **x** (≡force vector **f**). Because vector **s** operates as a scale factor to network *G*, in the extent it is defined by relation (8) (i.e. it escalates the weights matrix *W* to be equal with the stiffness tensor *K*), we can name the vector **s**(**x**,**y**)≡**s**_**x**,**y**_ as “*stiffness scale-factor of variables* **x**,**y**”. Overall, the previous pair-wise approach proposes a method for correlating, in a cause-effect context, two node-variables **x** and **y** of a complex network.

### 2.3. Implementation framework: The global inbound tourism network

The proposed stiffness-based computational approaches are evaluated in comparison with the existing measures of [16], in a real-world application context. For the implementation of this procedure, the strongest connected component (*G*_s_) of the global inbound tourism network (GTN) is used, as it was modeled by the authors of [19]. In particular, the GTN_s_ is modeled in the *L*-space representation (see [3]) into a directed weighted graph *G*(*V*,*E*), where nodes represent tourism destinations countries and links represent annual tourism flows from the country of origin to the country of destination. The strongest connected component was chosen to participate in the analysis instead of the whole GTN in order the adjacency of this sub-network’s not to be singular. Therefore, GTN_s_ was chosen to demand all computations to be possible. The GTN_s_ consists of 17 countries worldwide, as it is shown in Table 1.

**Table 1:** Countries included in the strongest connected component GTN_s_ of the global inbound tourism network*.

**Rank**	**Label**	**Country**	**Rank**	**Label**	**Country**
1	AUS	Australia	10	KOR	Korea, Rep.
2	AUT	Austria	11	MEX	Mexico
3	BEL	Belgium	12	NLD	Netherlands
4	CAN	Canada	13	NZL	New Zealand
5	FRA	France	14	ESP	Spain
6	DEU	Germany	15	CHE	Switzerland
7	IRL	Ireland	16	GBR	United Kingdom
8	ITA	Italy	17	USA	United States
9	JPN	Japan			

*. As modeled by the authors of [19].

**Fig 2:**
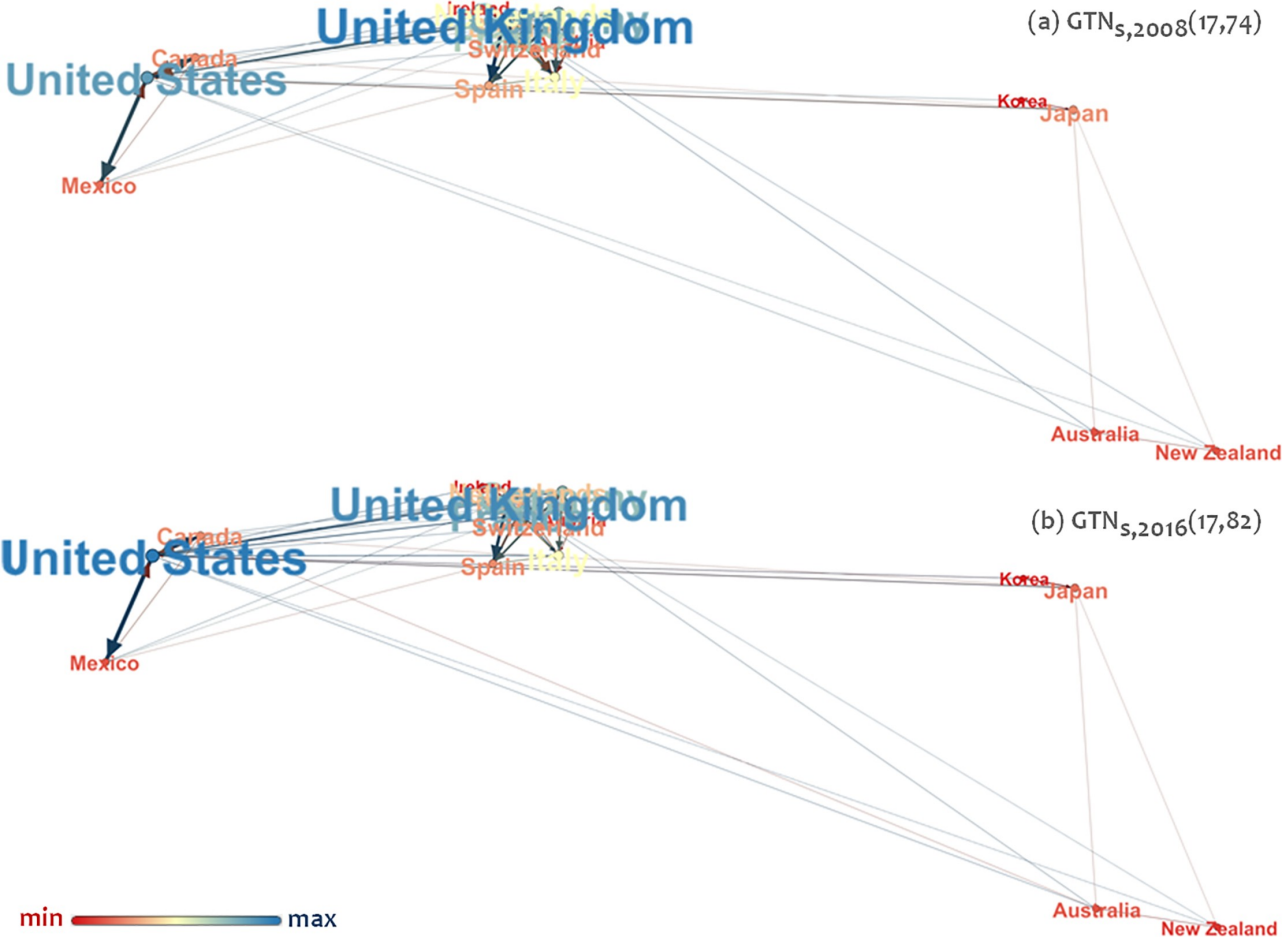
The strongest connected component (GTN_s_) of the global inbound tourism network (GTN) [19], shown in geo layout [24] embedding (a) for the year 2008 and (b) for the year 2016. Nodes express tourism destination countries, links express tourists’ arrivals from country (node) *i* to node *j*, and node size and coloring are proportional to node degree (undirected). Data extracted from [20].

Two versions of the GTN_s_ are considered in the analysis, as shown in Fig 2; the first refers to the year 2008 and the second to the year 2016. These two versions are constructed on data extracted from references [20–23].

The node-variables (attributes) considered in the analysis refer to the network topology and to the socioeconomic framework of GTN_s_. In particular, node-variables participating in the analysis are shown in Table 2. The socioeconomic attributes are chosen from the literature [19,20–23,25] according to their relevance in the determination of the phenomenon of tourism demand.

**Table 2:** Variables considered in the analysis.

Node-variable (attribute)	Symbol	Description	Reference
a. Variables of network topology			
Degree	*k*	Number of connections adjusted to a node (undirected).	[26]
Node (incoming) strength	STR	Sum of weights of the incoming connections adjusted to a node (directed).	[3]^*^
Clustering coefficient (local)	*C*	Defined by the fraction of number of a node’s connected neighbors E(i) (i.e. the number of triangles) to the number of the total triplets (equal to *k*_i_(*k*_i_–1)) shaped by this node. It expresses the tendency of nodes to cluster with their neighbors or the probability of meeting linked neighbors around a node (undirected).	[3]^*^
Betweenness centrality	CB	Defined by the fraction of all shortest paths in the network including a given node, to the total number of all shortest path in the network (undirected).	[27]^*^
Closeness centrality	CC	Defined by the inverse average distance of the shortest paths from a given node to all the others in the network. It expresses node accessibility (undirected).	[27]^*^
Eigenvector Centrality	EIG	Spectral measure computed on the eigenvectors of the adjacency matrix and it measures the influence of a node (undirected).	[26]^*^
b. Socioeconomic variables**			
Population	POP	Number of nationals present in, or temporarily absent from a country, and aliens permanently settled in a country.	[21]
Gross domestic product	GDP	Defined by the expenditure on final goods and services minus imports.	[22]
Unemployment	UNEMP	The share of the labor force that is without work but available for and seeking employment (% of total labor force).	[23]

*. Computed on data from [20]

**. Node (incoming) strength (STR) can be included also in this group

The dynamics of the GTN were computed for two time-snapshots *t*_*a*_ = 2008 and *t*_*b*_ = 2016. Therefore, all formulas defined previously are computed on the differences d*x*_*i*_ = *x*_*i*_(2016)–*x*_*i*_(2008). Measures, which are computed using formulas (2):(5) and (10), refer to a certain attribute (node-variable) *X* extracted from Table 2. For instance, variable **f**(*k*) expresses a force-alike vector computed on the attribute of degree (*k*), variable **d**(POP) expresses a displacements-alike vector computed on the population (POP) attribute, variable **s**(EIG) expresses a scale-factor vector computed for variables **x** = EIG_2008_ and **x** = EIG_2016_, and variable **s**(*k*,EIG) expresses a scale-factor vector computed for variables **x** = *k* and **x** = EIG, for the same reference-year. Finally, the analysis is implemented using the Spearman’s (rank) correlation coefficient *r*_*s*_ [28]. In particular, *r*_*s*_ is computed using the standard formula of the Pearson’s correlation coefficient, according to the relation:

$$\rho =\frac{\mathrm{cov}\left(x,y\right)}{s_{x}s_{y}},$$ (11)

where cov(**x**,**y**) is the covariance of (vector) variables **x** and **y**, and *s*_*i*_ is the sample standard deviation of variable *i* = **x**,**y**. However, the Spearman’s correlation coefficient *r*_*s*_ is computed on the value-ranks variables rnk(**x**) and rnk(**y**) instead of on these variables’ values. Ranks are produced by ascending sorting of values within variables **x** and **y**. Therefore, it stands that *r*_*s*_ = *ρ*(rnk(**x**), rnk(**y**)) and that the Spearman’s coefficient of correlation ranges within the interval [–1,1], describing a perfect linear relation (positive or negative) in cases where |*r*_*s*_| = 1.

## 3. Implementation

### 3.1. Measuring the cause-effect (force-displacements) vectors for two time-states of GTN

By assuming that the GTN is a structured-framework-alike network (i.e. it behaves similarly with a structured framework), we can compute the forces applied to network nodes, for a measurable deformation, and, vice versa, the deformations (displacements) caused by known forces. First, for computing the forces-alike vectors **f**(*X*), we define as displacements the differences:

$$d_{\mathrm{observed}}\left(X\right)=\left[x_{i}\left(2016\right)-x_{i}\left(2016\right)\right]{}_{n\times 1},$$ (12)

where *x*_*i*_ is the *i*-th element of variable **x** belonging to the set {*k*, STR, C, CB, CC, EIG, POP, GDP, UNEMP} of Table 2. Based on the observed displacements, we compute the forces-alike vectors **f**(*X*) according to relation (4). Next, for computing the displacements-alike vectors **d**(*X*), we define as forces the differences:

$$f_{\mathrm{observed}}\left(X\right)=\left[x_{i}\left(2016\right)-x_{i}\left(2016\right)\right]{}_{n\times 1},$$ (13)

which are computed on variables shown in Table 2. Based on the observed forces-alike variables, we compute the displacements-alike vectors **d**(*X*) according to relation (5). Also, we compute the impact and stability node-variables according to relations (2) and (3), for the set of available node-variables of Table 2. Finally, we apply pair-wise correlations *r*_*s*_ = *ρ*(rnk(**x**), rnk(**y**)) on the set of the 36 available node variables, which were computed according to relation (11).

**Fig 3:**
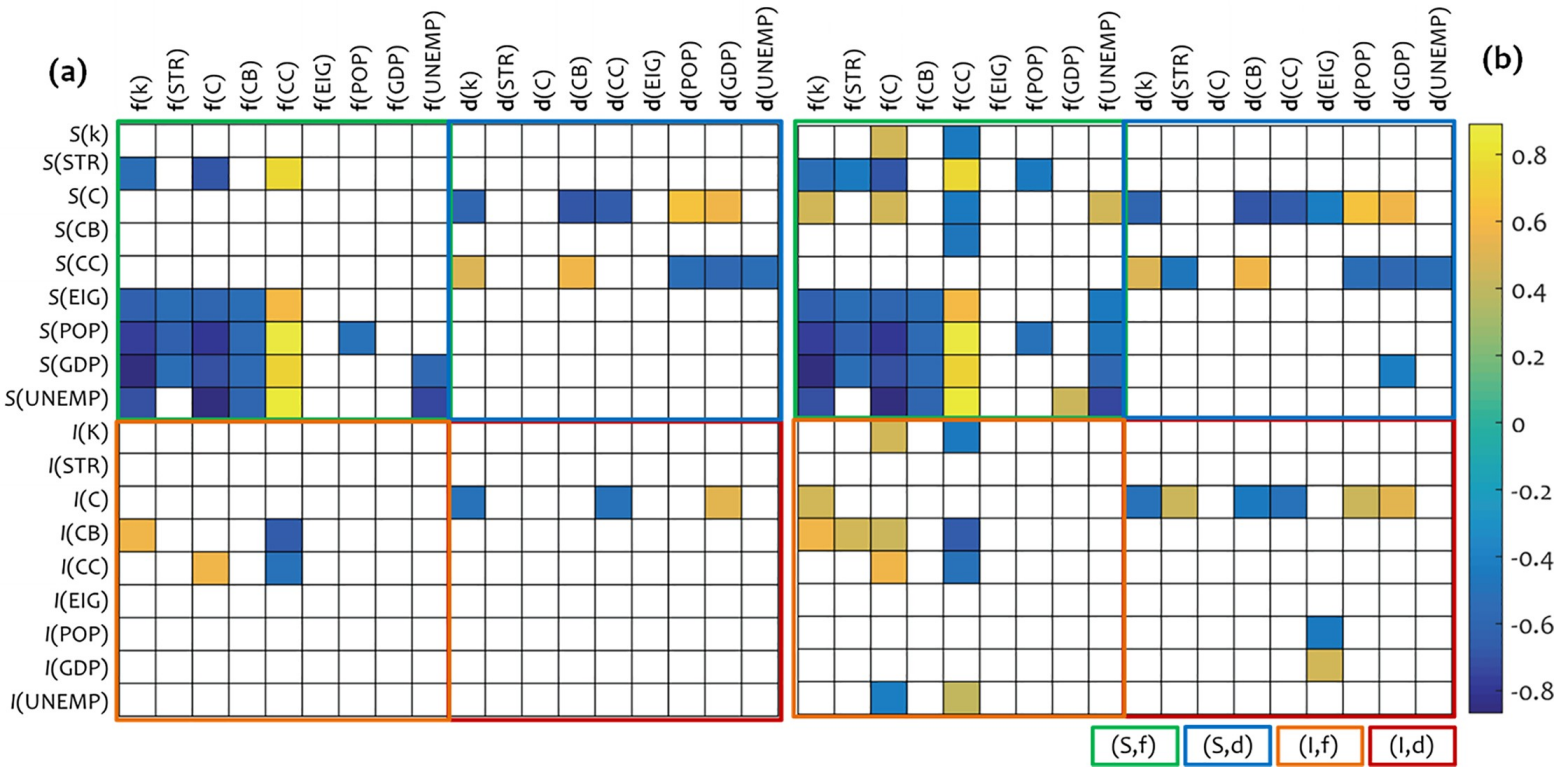
Spearman’s non-parametric correlations, (a) at 5% level of significance and (b) at 10% level of significance, between forces-alike **f**(*X*) and displacements-alike **d**(*X*) variables, stability *S*(*X*), and impact *I*(*X*). The **f**(*X*) and **d**(*X*) variables were computed using the proposed formulas (4) and (5), respectively, while *S*(*X*) and *I*(*X*) were computed using formulas (2) and (3), respectively. Symbols (*X*) represent attributes *X*∈{*k*, STR, C, CB, CC, EIG, POP, GDP, UNEMP}, according to which each variable is computed. Only significant correlation coefficients are shown.

The (significant-only) results of the analysis are shown in Fig 3, where we can observe that the forces-alike vector variables **f**(*X*) generally appear to be correlated with the stability variables *S*(*X*). This observation does not concern pairs (**f**, *I*) of the same attribute *X*, but systematic (almost universal) correlations appearing between the group of force-alike variables Φ_*T*_ = {**f**(*k*), **f**(STR), **f**(C), **f**(CB), **f**(CC)}, the variable *S*(EIG), and the group of socioeconomic variables Σ_*S/E*_ = {*S*(POP), *S*(GDP), *S*(UNEMP)}. The majority of these correlations are negative, illustrating an inverse analogy between the force-alike variables (**f**) and the variables of stability (*S*). This outcome is reasonable, because **f**-variables are conceptually related with the cause of deformation in the network, whereas *S*-variables are related with the resistance [16] of the network. An exemption from this pattern of negative analogy is the variable **f**(CC) of the clustering coefficient (CC) attribute, which is positively correlated with *S*(EIG) and Σ_*S/E*_, obviously due to the inverse definition if CC (see Table 2). Overall, the significant correlations observed for pairs (Φ_*T*_, *S*(EIG)) and (Φ_*T*_, Σ_*S/E*_) imply that the force-alike concept of network stiffness is effective to be used as a measure of network topology because it includes at the same time information related to node stability, to the spectral configuration of the network due to its relevance with eigenvector centrality [5], and to the socioeconomic framework of the GTN_s_. This can be especially useful for multidisciplinary research in complex networks, because the force-alike measures of stiffness capture information that is not restricted to the reference attribute *X* but they include broader information about the spectral and socioeconomic configuration of the network.

As far as the displacements-like variables are concerned, we can observe that **d**(*X*)-based correlations do not appear as systematic as those with the **f**(*X*)-variables. However, we can observe a trend of **d**(*X*)-based correlations to include information specialized to the *S*(C) and *S*(CC) variables. Despite the lack of universality, the (*S*, **d**) correlation-block shapes a supplementary picture in comparison with the correlations observed for the (*S*, **f**), a fact showing that the conceptual framework connecting the force-alike and displacements-alike stiffness-measures with the measures of impact and stability appears consistent.

A final remark about **f**(*X*)-based and **d**(*X*)-based correlations is that they appear in their vast majority indifferent to the impact-based variables. This observation seems reasonable because the measure of impact has intrinsic configuration, capturing nodes’ response to perturbations [16], and thus is more trend-based (or strain-based), in comparison with the **f**-alike and **d**-alike stiffness-measures which have an external, measurable, cause-effect, configuration.

### 3.2. Measuring the scale factor vector for two time-states of GTN

At next, we calculate the scale factors **s**(*X*) for the variables shown in Table 2 and we apply the same as previously correlation analysis between **s**(*X*) and *I*(*X*) and *S*(*X*). This time the scale-factor vectors **s**(*x*) are computed on a single attribute *X*, according to the formula:

$$s\left(X\right)=s\left(x_{2008},x_{2016}\right)|x_{2008}=\left(\left(\mathrm{diag}\left(s\left(X\right)\right)\right)\cdot W\right)\cdot x_{2016},$$ (14)

where we consider as forces-alike vector the node-variable of attribute *X* for the year 2008 and as displacements-alike vector the variable of the same attribute (*X*) for the year 2016. Computations are repeated accordingly for all attributes of Table 2. This approach produces scale-factor vectors **s**(*X*) describing the level to which a network should escalate so that a force-alike attribute *X* = **x**_2008_, observed for the year 2008 for the GTN_s_, to cause displacements-alike effects equal to the values *X* = **x**_2016_ of the same attribute observed for the year 2016. Within this context, we apply pair-wise correlations between the produced **s**(*X*), *I*(*X*), and *S*(*X*).

**Fig 4:**
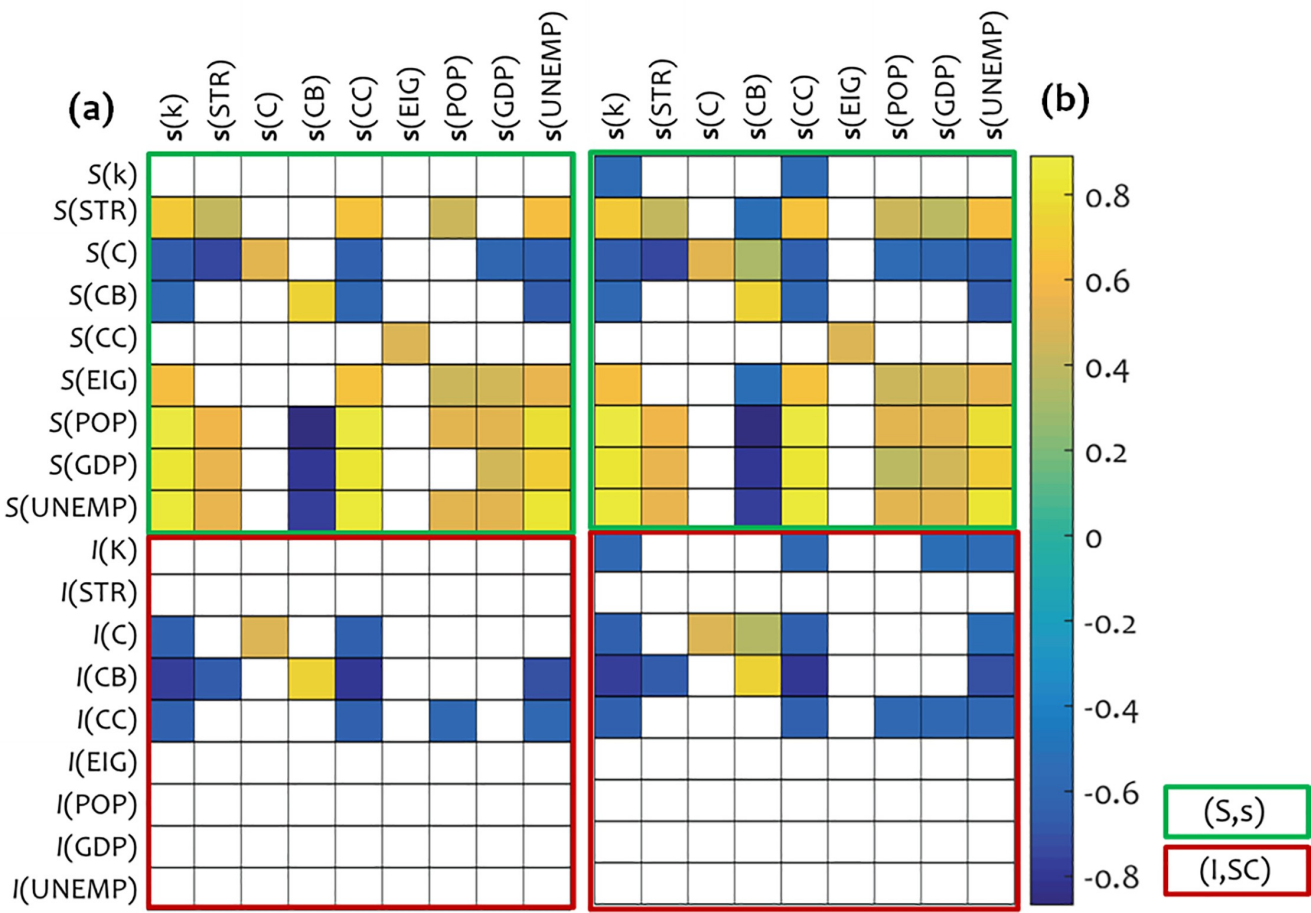
Spearman’s non-parametric correlations, (a) at 5% level of significance and (b) at 10% level of significance, between forces-alike **f**(*X*) and displacements-alike **d**(*X*) variables, stability *S*(*X*), and impact *I*(*X*). The **s**(*X*) variables were computed using the proposed formula (10), while *S*(*X*) and *I*(*X*) were computed using formulas (2) and (3), respectively. Symbols (*X*) represent attributes *X*∈{*k*, STR, C, CB, CC, EIG, POP, GDP, UNEMP}, according to which each variable is computed. Only significant correlation coefficients are shown.

The (significant-only) results of the scale-factor correlation analysis are shown in Fig 4, where we can observe a general inverse picture in comparison with this that was shaped by the (**f**-based and **d**-based) analysis of the previous sub-section. In particular, in the (*S*,**s**) correlation-block, we can observe systematic correlations between the group of the scale-factor variables X_*T*_ = {**s**(*k*), **s**(STR), **s**(CB), **s**(CC)}, the variable *S*(EIG), and the Σ_*S/E*_ group, which are inverse in comparison with those of Fig 3. An interesting observation within the (*S*,**s**) correlation-block is that now the **s**(CB) variable shows a negative analogy instead of the **f**(CC) variable in Fig 3. Based on the definitions of these two attributes [26], we can observe that betweenness centrality has more intrinsic topological concept (it is a shortest-path-based measure counting frequencies) than closeness centrality expressing accessibility. Another interesting remark is that the **s**(C) is a variable correlated just with the variables of its own attribute (*S*(C) and *I*(C)).

Finally, in Fig 4, we can observe a considerable number of correlations between scale-factor **s**(*X*) and impact-based variables included in the set {*I*(C), *I*(CB), *I*(CC)}. Despite the lack of universality, this implies that the scale-factor variables **s**(*X*) have more intrinsic configuration than the force-alike **f**(*X*) and displacements-alike **d**(*X*) variables shown in Fig 3. This is consistent with the way that **s**(*X*) is defined in relations (7),(8), and (10), according to which the measure of stiffness scale-factor has more spectral configuration because is being applied to the connectivity weights matrix *W* of the network and thus it operates indirectly in relation (4).

Overall, the correlations observed between the **s**(*X*) and impact-based and stability-based variables imply that the scale-factor concept of network stiffness can be also effective to be used as a measure of network topology. This is because, first, it includes inverse information to this extracted from Fig 3 and thus it has a supplementary utility to the force-alike and displacements-alike concept of network stiffness. Second, it includes additional impact-related information which complies with the spectral (and thus more intrinsic) configuration being evident by the way this measure is defined. Finally, an extra advantage of the scale-factor measure of stiffness, which is not possible to capture by the measures of impact and stability, is its potential to be used as a measure for correlating, in a cause-effect context, two node-variables **x** and **y** of a complex network.

## 4. Limitations and addresses of further research

This paper developed a conceptual analogy between a structured framework and a complex network aiming at considering the notion of stiffness from structural engineering as a topological property of complex networks. This approach sets limitations which are basically related with the introductory nature of the study. Therefore, regardless of how well is being documented, the analogy developed between a structured framework and a complex network should be further tested in more complex network applications. For instance, researchers who are activated in the fields of polymer and biological networks, where the structural-engineering-based concept of stiffness was first used, may contribute to the further evaluation of the proposed topological conceptualization of stiffness, based on literal data that do not necessitate the assumption of analogy between a structured framework and a complex network. However, provided that the conceptual analogy introduced in this paper appears fruitful in capturing dynamics in a spatio-socioeconomic and of immaterial nature network (i.e. the network of global tourism flows), then further applications about the network dynamics utilizing this new concept as a topological measure in complex networks are necessary. Especially, the researchers who are activated in the fields of network dynamics, of vulnerability, of information spreading and cascading, and of virus and disease spreading in complex networks can find many reasons to be motivated using this concept in their researches.

Despite the conceptual framework, one technical limitation about the proposed approach regards the inability to compute the displacements-alike vector according to relation (5) when the weights matrix of the complex may result to a zero determinant. This can happen, for instance, when the network is disconnected (i.e. it includes more than one components) or when it has a connectivity matrix of ones (e.g. it is a complete graph), namely *W*^*n*×*n*^ = [1]. Being the GTN a disconnected network restricted this paper to implement the analysis using the strongest connected component instead of the total network. However, avenues for further research suggest developing methods of repairing insufficient connectivity of the stiffness matrix similarly to the rationale of methods of repairing insufficient connectivity for the computation of centrality indices (see [27]).

Overall, the attempt of this paper to broaden the concept of network stiffness from just a material property into a property of network topology seems to set any emerging limitation to an upgrade-potential through further research. This potential provides added value to the proposed conceptual approach and serves the purpose of multidisciplinary demand in NS.

## 5. Conclusions

This paper showed that, by assuming that a complex network behaves similarly with a structured framework, it is possible to use the classic measure of stiffness from structural engineering to network science and thus to consider the new concept of network stiffness as a topological property in complex networks. The paper conceptualized three different aspects of network stiffness, the force-alike, the displacements-alike, and the scale-factor vectors in order to be used in complex network analysis. The implementation of the proposed measures in a real-world network and their evaluation in comparison with propagation measures in the analysis of network dynamics showed that stiffness can produce interesting insights to network science.

In particular, the correlation analysis applied between the available variables showed that the force-alike concept (**f**) of network stiffness is effective to be used as a measure of network topology because it includes at the same time information related to node stability, to the spectral configuration, and to the socioeconomic framework of the network. Also, it showed that the displacements-alike concept (**d**) of network stiffness is supplementary to the force-alike one, which both may shape an extrinsic picture of the network-dynamics’ framework. Finally, the analysis showed that the scale-factor concept (**s**) of network stiffness is equipped with a more intrinsic functionality, which due to its spectral configuration it integrates the previous two stiffness-based measures because except with the measure of stability it is also correlated with node impact. An extra advantage about the scale-factor stiffness concept, which cannot be captured by any other among the examined measures, is its potential to be used as a measure for correlating, in a cause-effect context, any pair of node-variables of a complex network.

Overall, the analogy of assuming that a complex network behaves similarly with a structured framework showed potentials to provide added value in the multidisciplinary research of complex networks, as the case study of the spatio-socioeconomic global inbound tourism network has shown. In spite of detecting significant correlations between the proposed concepts and the existing measures of propagating network dynamics, the overall approach instructs more for a supplementary rather than a single use of all these measures (both existing and proposed) in order to shape a more spherical picture about network dynamics. As being evident, stiffness can suggest a promising concept for network science, capable in providing interesting insights and to motivate for further research.
